# XBP1 negatively regulates CENPF expression via recruiting ATF6α to the promoter during ER stress

**DOI:** 10.1186/s12935-020-01553-9

**Published:** 2020-09-22

**Authors:** Tao Shen, Yan Li, Shuang Liang, Zhiguang Chen

**Affiliations:** 1 Department of Orthopedics, Shengjing Hospital of China Medical University, No. 36, Sanhao Street, Heping District, Shenyang, 110004 People’s Republic of China; 2 Department of Cell Biology, Key Laboratory of Cell Biology, Ministry of Public Health and Key Laboratory of Medical Cell Biology, Ministry of Education, China Medical University, Shenyang, 110122 People’s Republic of China; 3 Sanford Burnham Prebys Medical Discovery Institute, La Jolla, CA 92037 USA; 4 Department of Laboratory Medicine and Pathology, University of Minnesota, Minneapolis, MN 55455 USA

**Keywords:** ER stress, CENPF, Expression regulation, XBP1, ATF6α

## Abstract

**Background:**

Centromere protein F (CENPF) is a key component of the kinetochore complex involved in mitosis, cell differentiation and cellular response to stresses. However, the alteration of CENPF in response to endoplasmic reticulum (ER) stress has not been well described. In the present study, we investigate CENPF regulation in response to ER stress.

**Methods:**

Quantitative real-time polymerase chain reaction and western blotting were used to determine CENPF expression under ER stress. Luciferase activity analysis was performed to investigate the promoter regions contributing to CENPF transcription in response to TG. Chromatin immunoprecipitation (ChIP) and ChIP Re-IP assays were used to determine if X-box binding protein 1 (XBP1) and/or activating transcription factor 6α (ATF6α) bind in the CENPF promoter region. Cell apoptosis and proliferation were analyzed using TUNEL, cell growth and clonogenic assays.

**Results:**

CENPF expression is dramatically reduced under ER stress induced by thapsigargin (TG), brefeldin A (BFA), or tunicamycin (TM) and this downregulation of CENPF expression was dependent on XBP1 and ATF6α. Luciferase activity analysis of the truncated CENPF promoter indicates that regions from bases − 679 to − 488 and from − 241 to − 78 in the CENPF promoter were sensitive to TG treatment. Additionally, ChIP and ChIP Re-IP assays reveal that XBP1 and ATF6α were assembled on the same regions of CENPF promoter. Notably, we identify two XBP1 binding sequences at positions − 567 and − 192, to which XBP1 binding was enhanced by TG. Finally, CENPF overexpression inhibits cell apoptosis and promotes cell proliferation in response to ER stress.

**Conclusion:**

In summary, these results demonstrate that ER stress plays a crucial role in CENPF expression, and XBP1 may up-regulate DNA-binding affinities after TG treatment to the promoter of CENPF. These findings may contribute to the understanding of the molecular mechanism of CENPF regulation.

## Background

Centromere protein F (CENPF), which is located on chromosome 1q41, encodes for a microtubule-associated protein involved in mitosis and cell differentiation [1]. CENPF was upregulated during the G2/M phase and accumulates to the kinetochore complex, facilitating microtubule attachment and chromosome segregation [1, 2]. A number of studies have reported that CENPF expression is overexpressed in several human malignancies including breast cancer [3], hepatocellular carcinoma [4], nasopharyngeal cancer [5], gastrointestinal stromal tumors [6], esophageal squamous cell carcinoma [7], and non-Hodgkin’s lymphoma [8]. Additionally, in some cases CENPF expression is associated with aggressive tumor phenotype and poor survival [3–5]. It was recently observed that CENPF and forkhead box M1 (FOXM1) cooperate together, acting as synergistic master regulators of malignancy in prostate cancer [9]. CENPF promotes breast cancer bone metastasis by activating PI3K-AKT-mTORC1 signaling [10]. The expression of CENPF has been detected in different cancers, however its expression pattern differs among various types of cancer. These diverse observations suggest that while CENPF could potentially be a therapeutic target and its role in tumorigenesis may depend on cell type and tumor environment.

The endoplasmic reticulum (ER) is a large and dynamic cellular structure that serves in various roles, including ensuring the correct folding of proteins residing within, maintaining of Ca^2+^ homeostasis and transiting along the secretory pathway [11–13]. Stress of the ER leads to the activation of an unfolded protein response (UPR) signaling pathway in order to prevent uncontrolled protein misfolding and restore the ER homeostasis [14, 15]. In mammalian cells, the UPR signaling pathway is coordinated by three main ER-proximal sensors that respond to increased levels of unfolded proteins: inositol-requiring protein 1α (IRE1α), PRKR-like ER kinase (PERK), and activating transcription factor 6α (ATF6α). The outcome of UPR activation increases protein folding, transport and ER-associated protein degradation (ERAD) in which the proteins in the ER are retro-translocated to the cytosol for proteasomal degradation, and decreases protein synthesis [14, 16]. Notably, ER stress pathway components are dysregulated in almost every human pathological disorder, including neurodegenerative, diabetes, metabolic disorders, inflammatory diseases and cancer [17, 18].

CENPF plays an important role in stress response resulting from DNA damage, mitotic stress, oxidative stress, and hypoxic. However, the linkage between ER stress and CENPF expression has not yet been described [19–21]. The purpose of the present study is to elaborate a possible mechanism, which brings about the changes of CENPF expression through an ER stress-mediated manner in human osteosarcoma cells. In this study, we have examined the effects of ER stress on CENPF expression in U2OS and MG-63 cells. Dramatic decreases in CENPF mRNA and protein were observed when cells were treated with different ER stress inducers. Furthermore, we discovered that XBP1 negatively regulated CENPF expression in response to ER stress. In addition, we also identified that two XBP1 binding motifs at positions − 567 and − 192 relative to the transcription start site, that were involved in the induction of CENPF promoter under ER stress. Our findings suggest that ER stress induces XBP1 and ATF6α binding, which may increase their DNA-binding affinity and inhibit the transcription activity of the CENPF gene.

## Methods

### Cell lines and reagents

Human osteosarcoma cell lines (U2OS and MG-63) were purchased from the American Type Culture Collection. The cell lines were maintained in Dulbecco’s Modified Eagle’s Medium (DMEM, GIBCO, Los Angeles, CA, USA) supplemented with 10% fetal bovine serum (FBS; Thermo Fisher Scientific, Inc.), and penicillin–streptomycin (Gibco; Thermo Fisher Scientific, Inc.) at 37 °C in a humidified atmosphere of 5% CO_2_. pGL3-Basic, pRL-TK and the Dual luciferase reporter assay system were purchased from Promega (Madison, WI, USA). Brefeldin A (BFA), thapsigargin (TG), and tunicamycin (TM) were obtained from Sigma-Aldrich (St. Louis, MO, USA).

### Plasmid construction and mutagenesis

The luciferase reporter plasmids used in this study were derived from pGL3-Basic. A series of 5’-deleted CENPF constructs were derived from pGL3- CENPF-promoter by PCR. The sense primers were 5’-AAGGTAAAGTCAGGGGGCTG-3’ (− 840), 5’-AGTGGGCTTCACGAAAAGCA-3’ (− 680), 5’-GTACTTAGCTTCTATGAGCC-3’ (− 488), 5’-GACTTTTGCGGAAAT3’ (− 242), and 5’-GTCTGAGTGCGCAGGCGCGG-3’ (− 78). The antisense primer was 5’-GGCGGGCTGGAGCCCAGAGT-3’ (+ 60). Mutations of p680m-luc (− 567: ATGA, underlined mutated bases) and p242m-luc (− 192: ATGA, underlined mutated bases) were generated using the Quickchange-XL Site-Directed Mutagenesis kit (Stratagene) using p680-luc and p242-luc as a template. p680d-luc (− 567: ACGT deletion) and p242d-luc (− 192: ACGT deletion) deletion constructs were generated with Q5 Site-Directed mutagenesis kit using p680-luc and p242-luc as a template. All constructs were confirmed by sequencing without coding frame shifts in the luciferase gene.

### Transient transfection and luciferase assays

U2OS cells were transfected as indicated using Lipofectamine™ 2000 (Invitrogen, USA) or JetPrime (Polyplus, France) according to the manufacturers’ protocols. pRL-TK plasmids containing the Renilla luciferase gene were used as internal controls to normalize transfection efficiency. After 24 h of transfection, the cells were incubated with 1 μM TG for an additional 24 h. Luciferase activities were determined with the Dual Luciferase Reporter assay system using a Lumat LB9507 luminometer (Bethold Technologies, Germany).

### Gene silencing

Negative control (non-silencing siRNA) or siRNA targeting the transcript of interest was transfected into U2OS cells using Lipofectamine™ 2000 (Invitrogen, USA). All the short-interfering RNAs were obtained from

Invitrogen, whereas shRNA vectors silencing for CENPF were purchased from Sigma.

### Western blot analysis

Cells were lysed using RIPA buffer (50 mM Tris–HCl [PH7.4], 1% NP-40, 0.1% sodium deoxycholate, 0.1% SDS, 150 mM NaCl, 1 mM EDTA, and phosphatase and protease inhibitors) and the concentration of protein was assessed using a bicinchoninic acid assay (Beyotime Institute of Biotechnology). Lysates were sonicated, centrifuged, and subjected to sodium dodecyl sulfate (SDS)-polyacrylamide gel electrophoresis (PAGE). Proteins were transferred onto a nitrocellulose membrane (Osmonics Inc., USA). Membranes were subsequently probed with primary anti-CENPF (1:1000; cat. no. ab5; Abcam) or anti-α-Tubulin (1:500; cat. no. sc-5286; Santa Cruz) by overnight incubation at 4 °C. Imaging of immunoblots were performed with a LICOR system using respective fluorescence antibody: IRDye^®^ 800CW Donkey anti-Mouse IgG Secondary Antibody (1:15,000; cat. no. C50422-04; LICOR).

### Reverse transcription and quantitative real-time PCR

Total RNA was extracted extracted from cells using a total RNA miniprep RNeasy Mini Kit (Sigma) and digested with DNase I. cDNA was synthesized using oligo(dT) and random primers (AB Bioscience, USA) for SYBR Green qPCR analysis. Real-time PCR was performed on a LightCycler (Roche). PCR was carried out as follows: 95 °C for 10 min; 40 cycles of 95 °C for 10 s and 60 °C for 40 s. Primer sequences for detection of CENPF mRNA expression were synthesized as 5′-ACCTTCACAACGTGTTAGACAG-3′ (sense) and 5′-CTGAGGCTCTCATATTCGGCA-3′ (anti-sense). The primers used for analysis of 18S rRNA used as internal control were: 5′-GTAACCCGTTGAACCCCATT-3′ (sense) and 5′- CCATCCAATCGGTAGTAGCG-3′ (anti-sense). Triplicate biological samples were used for the qPCR analysis. Gene expression level was normalized to that of 18 s rRNA. Relative gene expression was analyzed using the 2-ΔΔCq method [22].

### ChIP (chromatin immunoprecipitation) and ChIP Re-IP

The ChIP and ChIP Re-IP assays have been described previously in detail [23, 24]. Briefly, U2OS cells were treated with or without 1 μM TG for 24 h and crosslinked with 1% formaldehyde for 20 min at 37 °C. Cells were lysed and sonicated, and extracted were precleared with protein A/G magnetic agarose overnight at 4 °C. Following immunoprecipitation with anti-XBP1(cat. no. ab28715, Abcam) or anti-IgG antibodies, protein complexes were washed in turn with low salt, high salt, lithium chloride buffer and TE buffer. After four washes, crosslinking of the protein/DNA complex was reversed. DNA was then purified using a spin column, and subjected to qPCR analysis. Primer pairs for CENPF promoter (− 679–− 488) were: 5′-AGTGGGCTTCACGAAAAGCA-3′ (sense), and 5′-TTGAGGAAAGTATTATCCT-3′ (antisense) and CENPF promoter (− 241 ~ − 78) were: 5′-GACTTTTGCGGAAAT-3′ (sense), and 5′-GCCGCGTCTGATTGGCCCTT-3′ (antisense).

Primer pairs for upstream and downstream were: 5′-GTTACTAGGGATGCAAAAAT-3′ (upstream sense), 5′-TCATTAGACTGTTCCTGCAG-3′ (upstream antisense), 5′-GGATTGGTCCGCAGCTACTTA-3′ (downstream sense), and 5′-CTTGCTCTCGGGGACGGGAA-3′ (downstream antisense). Ct values of control and treated conditions were normalized to the corresponding input values.

For ChIP Re-IP, complexes were eluted from the primary immunoprecipitation by incubation with 10 mM DTT at 37 °C for 30 min and diluted in buffer [1% Triton X-100, 2 mM EDTA, 150 mM NaCl, 20 mM Tris/HCl (pH 8.1)] followed by reimmunoprecipitation with the second antibodies. The qPCR primers for ChIP Re-IPs were the same as those for ChIP assays.

### TUNEL assay

Apoptotic cells were analyzed using the ApopTag Peroxidase In Situ Apoptosis Detection Kit (Chemicon, USA) according to the instructions of the manufacturer.

### Cell growth and clonogenic assay

For cell growth assay, U2OS cells (2 × 10^5^) were seeded in triplicate in 60-mm plates and allowed to grow for 24 h. Cells were then treated with TG (1 μM) for 24 h before harvesting. Viable cells were counted following trypan blue staining, and quantified using ImageJ software (National Institute of Health).

For the clonogenic assay, 6-well plates coated with bottom agarose prepared with 0.4% agarose in DMEM plus 10% FBS. U2OS cells, stably transfected with plasmids were added in top agar prepared with 0.4% agarose in DMEM plus 10% FBS and subsequently covered with DMEM plus 10% FBS and 1% streptomycin (Invitrogen) and grown for 7–10 days. Plated were fixed overnight with 4% PFA (Thermo Fisher Scientific) followed by stain 1 h at RT with crystal violet. Washes were performed with water until colonies could be visualized. The colonies were quantified using ImageJ software (National Institute of Health).

### Statistical analysis

Results represent the mean ± s.e.m. from three experiments, where appropriate representative results were depicted. Two groups were compared using the two-tailed t-test for parametric data or the Mann–Whitney U test for non-parametric data. Multiple groups were compared using one-way ANOVA. We defined statistical significance as *P < 0.05, **P < 0.005, ***P < 0.001, and ****P < 0.0001. All statistical analyses were performed using GraphPad Prism (GraphPad Software, Inc., San Diego, CA).

## Results

### ER stress results in a decrease of CENPF expression in human osteosarcoma cells

**Fig. 1:**
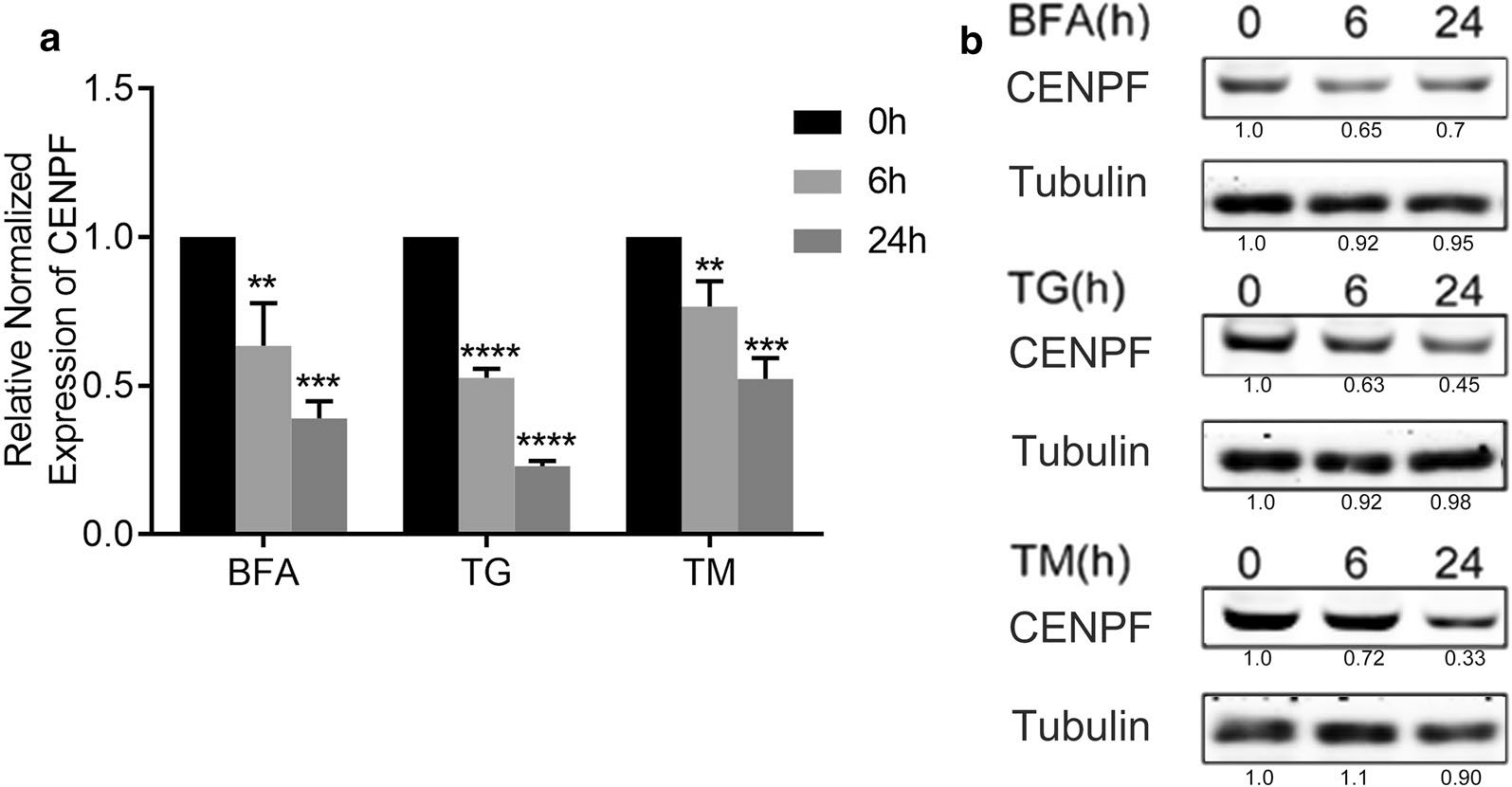
CENPF expression is downregulated in osteosarcoma U2OS cells in response to ER stress. **a** U2OS cells were treated with DMSO vehicle (control), BFA (1 μg/ml), TG (1 μM) or TM (2.5 μg/ml) for 0, 6, or 24 h. The CENPF mRNA levels were quantified by real-time reverse transcription-PCR (q-RT-PCR) and normalized to 18S RNA. **b** Changes in CENPF protein levels induced by BFA, TG and TM treatment in U2OS cells. CENPF and α-tubulin levels were determined by western blot analysis. Data are representative of 3 independent experiments. Data are presented as mean ± s.e.m. *P < 0.05, **P < 0.005, ***P < 0.001, ****P < 0.0001

To investigate the effect of ER stress on CENPF expression, CENPF mRNA level was analyzed using real-time PCR in human osteosarcoma cell lines U2OS and MG-63 cells with different ER stress inducers. As shown in Fig. 1a and Additional file 1: Fig. S1a, treatment of U2OS and MG-63 with three well-established ER stress inducers, namely BFA, TG or TM, resulted in marked and time-dependent decrease in CENPF mRNA levels. Consistent with the above observation, BFA, TG or TM also caused a decrease of CENPF protein level in U2OS and MG-63 cells (Fig. 1b and Additional file 1: Fig. S1b).

### ER stress-induced decrease in CENPF expression is dependent on XBP1 and ATF6α

**Fig. 2:**
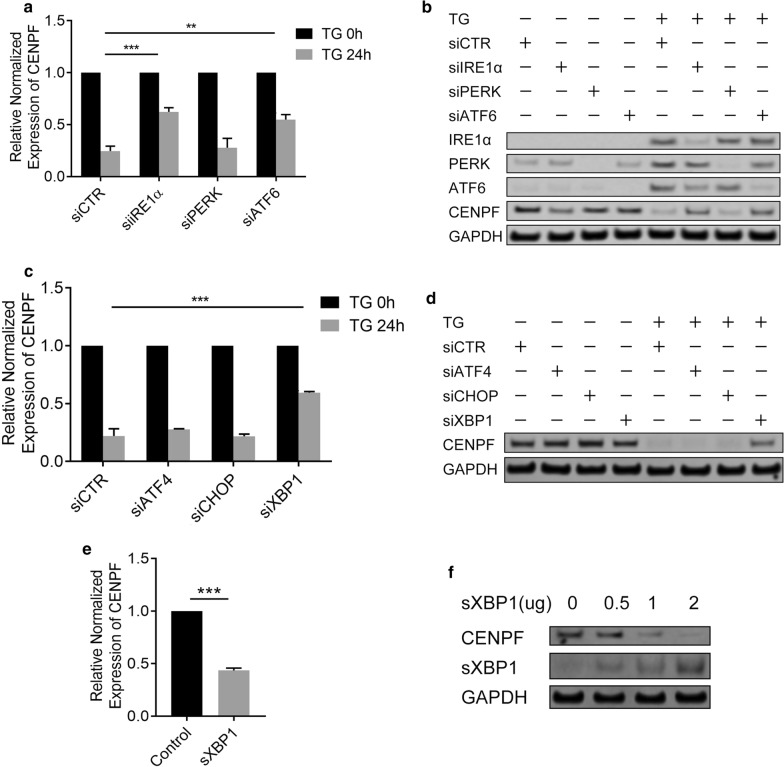
ER stress-induced decrease in CENPF expression is dependent on ATF6 and XBP1. **a** q-RT-PCR analysis of CENPF mRNA level in U2OS cells with or without transient knockdown of IRE1α, PERK and ATF6. **b** Western blotting analysis of CENPF, IRE1α, PERK and ATF6 protein level in U2OS cells with or without transient knockdown of IRE1α, PERK and ATF6. **c** q-RT-PCR analysis of CENPF mRNA level in U2OS cells with or without transient knockdown CHOP, XBP1 and ATF4. **d** Western blotting analysis of CENPF protein level in U2OS cells with or without transient knockdown of CHOP, XBP1 and ATF4. **e** 1 μg XBP1 spliced expression vector was transiently transfected into U2OS cells as indicated, and the mRNA level was quantified by q-RT-PCR analysis. **f** Different dose of XBP1 spliced expression vector was transiently transfected into U2OS cells as indicated, and CENPF protein level was estimated by western blot analysis. Data are representative of 3 independent experiments. Data are presented as mean ± s.e.m. *P < 0.05, **P < 0.005, ***P < 0.001, ****P < 0.0001

Three main pathways become activated during ER stress conditions, which involve IRE1α, PERK and ATF6, respectively. To determine whether these pathways are important for CENPF regulation, U2OS cells were transfected with 3 different siRNA species for IRE1α, PERK and ATF6 and then stimulated with the ER stress inducer TG for 24 h. While silencing of PERK had no effect in these studies, experimental reduction of IRE1α and ATF6*α* expression diminished ER stress-induced decrease in CENPF expression (Fig. 2a, b). Furthermore, we investigate which key transcription factors might be involved in CENPF regulation. Furthermore, we investigate which key transcription factors might be involved in CENPF regulation. XBP1 is a key transcription factor for the IRE1α signaling pathway, while ATF4 and CHOP are key transcription factors for PERK signaling pathway. We therefore transfected U2OS cells with different siRNAs of ATF4, CHOP and XBP1 and then stimulated overnight with r TG. As shown in Fig. 2c, d and Additional file 2: Fig. S2 reducing XBP1 mRNA and protein levels dramatically reversed ER stress-induced downregulation of CENPF expression. To confirm this result, we found spliced XBP1 overexpression decreased levels of CENPF mRNA and protein (Fig. 2e, f). Our results suggest XBP1 contributes to the ER stress-induced decrease in CENPF expression.

### Effect of ER stress on transcriptional activity of the CENPF promoter

**Fig. 3:**
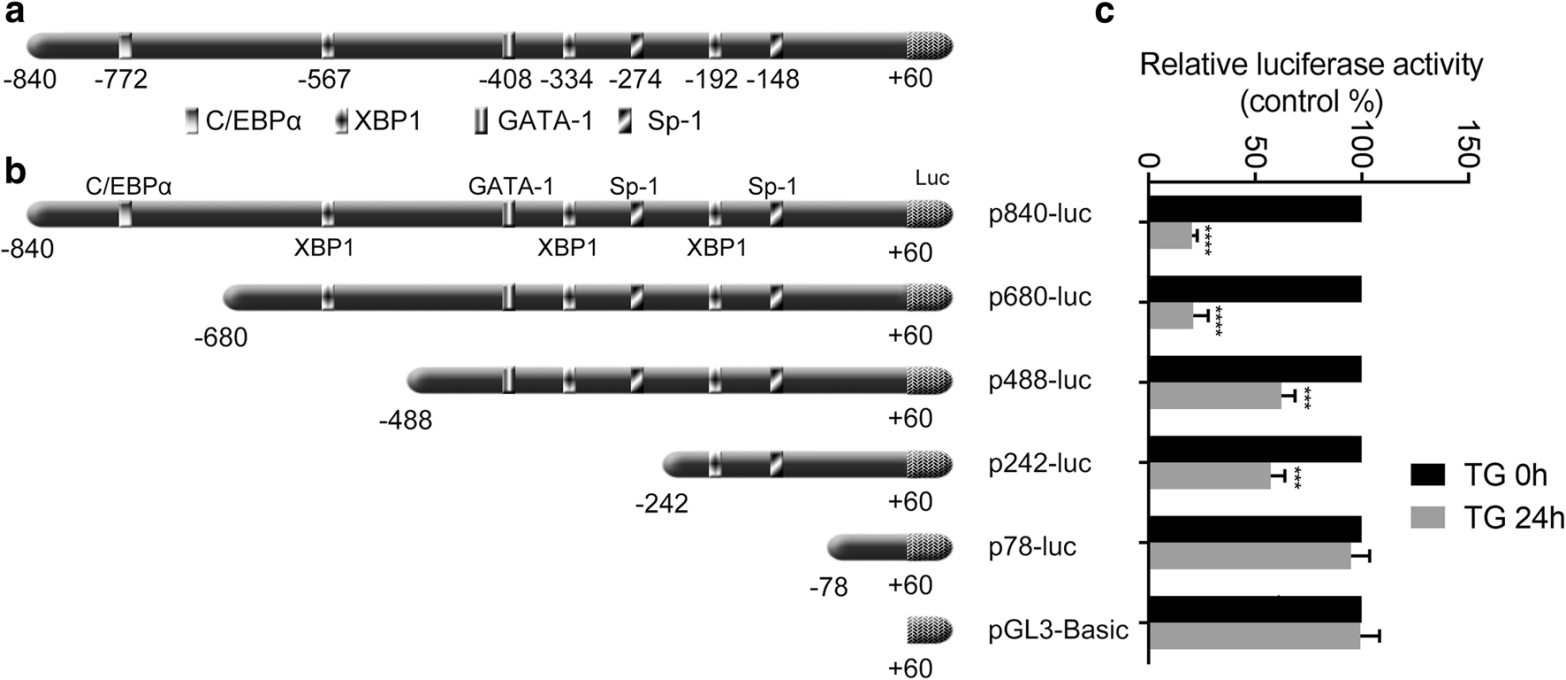
Transient transfection analysis of CENPF gene promoter constructs. **a** Using TRANSFAC-TESS and Match™ on-line softwares, we identified potential transcription factor binding motifs in the CENPF promoter. **b** Schematic representation of a series of 5′-deletion CENPF promoter luciferase constructs. Numbering is defined relative to the transcription start site. **c** Construction of a series of 5′-truncated CENPF promoters and their relative luciferase activities without (the white bar) or with (the black bar) 24 h TG treatment. Luciferase activities were determined and normalized to Renilla activity. Results are expressed as a percentage of the untreated control, which is taken as 100%. Data are representative of 3 independent experiments. Data are presented as mean ± s.e.m. *P < 0.05, **P < 0.005, ***P < 0.001, ****P < 0.0001

To continue investigating the underlying molecular mechanisms that regulate the CENPF gene expression during ER stress activation, we subsequently used TRANSFAC-TESS and Match™ searches to analyze the – 840 to + 60 nucleotide of the CENPF promoter with the. The CENPF promoter region did not contain known UPRE I (TGACGTCC/A) or UPRE-II (ATTGG-N-CCGCGT), however, it contain XBP1-binding sequences ACGT [25]. Moreover, the CENPF promoter region also contains binding motifs for several additional transcription factors that are regulated by ER stress, including C/EBPα at − 772, GATA-1 at − 408 and Sp-1 at − 274 and − 148 (Fig. 3a; Additional file 3: Fig. S3).

To delineate the potential roles of these transcriptional regulatory elements, we generated a series of luciferase reporter constructs containing 5′-flanking deletions of the CENPF promoter (Fig. 3b). These were transiently transfected into U2OS cells and cellular response to TG stimulation was examined. As shown in Fig. 3c, following 24 h exposure to 1 μM TG, the transcriptional activities of p840-luc and p680-luc were decreased to a lower level compared with those of other luciferase plasmids and pGL3-basic, while the transcriptional activities of p488-luc was increased compared with that of p840-luc and p680-luc. The transcriptional activities of p488-luc and p242-luc were decreased compared with that of p78-luc after TG treatment. These data imply that the promoter regions from − 679 to − 488 and from − 241 to − 78 contribute significantly to regulating CENPF transcription in response to TG. We therefore focused on the putative XBP1 binding sequences at − 567 and − 192 of the CENPF promoter.

### XBP1 and ATF6α cooperatively binds to the CENPF promoter region to regulate CENPF expression in human osteosarcoma cells under ER stress treatment

**Fig. 4:**
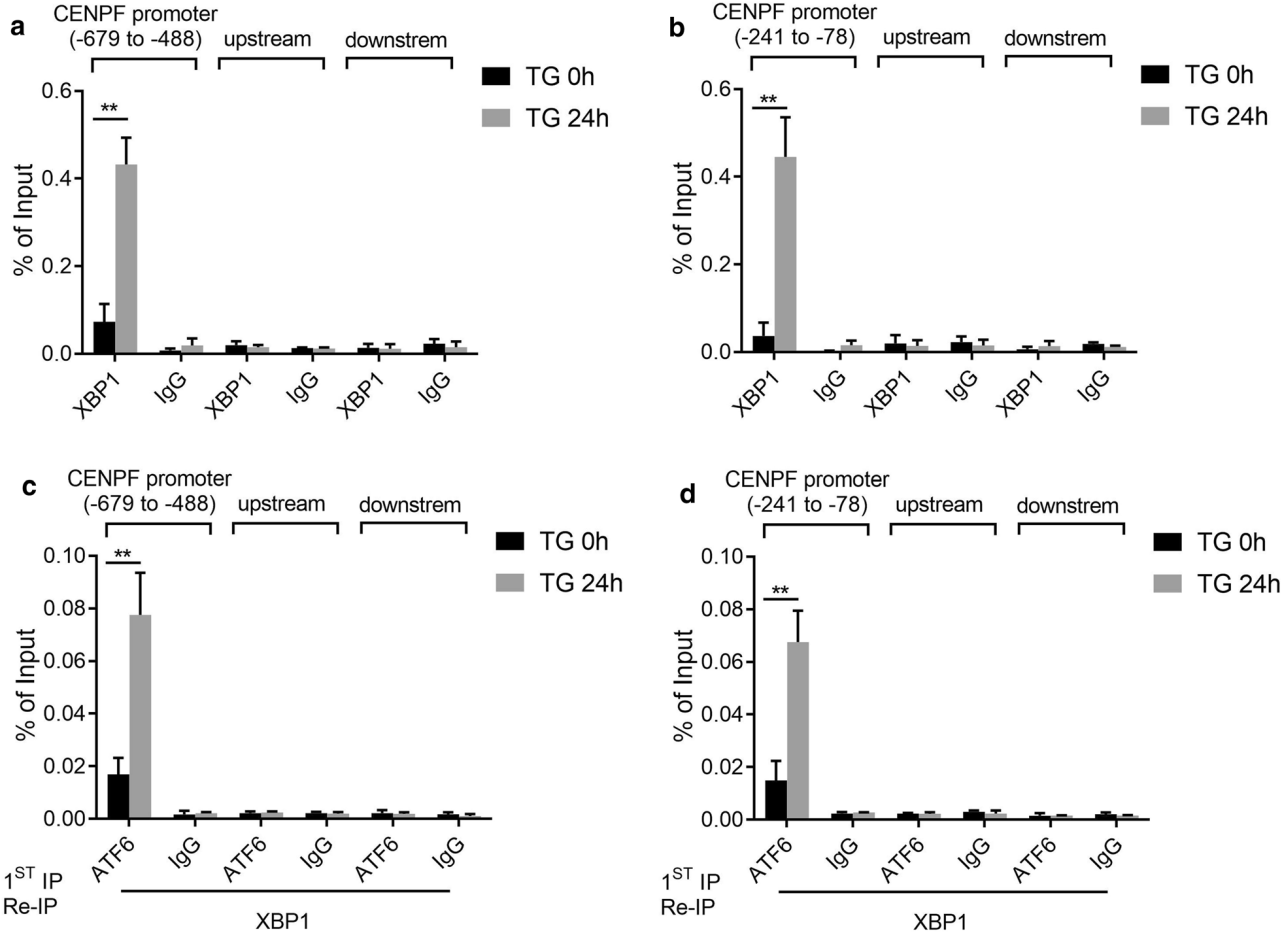
ChIP and ChIP Re-IP assays analysis of TG-induced binding of XBP1 and ATF6 to the CENPF promoter in vivo. **a** ChIP assay to examine binding of XBP1 to the CENPF promoter region (− 679 to − 488) by TG in vivo. **b** ChIP assay to examine binding of XBP1 to the CENPF promoter (− 241 to − 78) by TG in vivo. **c** ChIP Re-IP to examine whether XBP1and ATF6 were assembled on the same CENPF promoter region (− 679 to − 488). **d** ChIP Re-IP to examine whether XBP1and ATF6 were assembled on CENPF promoter region (− 241 to − 78). Data are representative of 3 independent experiments. Data are presented as mean ± s.e.m. *P < 0.05, **P < 0.005, ***P < 0.001, ****P < 0.0001

We next investigated whether XBP1 binds to the CENPF promoter and whether this binding is affected ER stress under in vivo conditions. Untreated and TG-treated cells were sonicated, and extracted were subjected to chromatin immunoprecipition with XBP1 antibody. This was followed by PCR analysis with primers specific for the CENPF promoter. As shown in Fig. 4a, b, binding of XBP1 was detected at the − 679 to − 488 and − 241 to − 78 regions of the CENPF promoter. Importantly, ER stress treatment resulted in a significant increase in the DNA-XBP1 binding affinity. These results demonstrate the functional association of XBP1 with the CENPF promoter.

The transcription factor XBP1 binds the promoter as homodimer or as heterodimer together with ATF6α [26]. To further examine whether XBP1 and ATF6α were assembled on the same region of promoter, ChIP Re-IP assays were carried out. The soluble chromatin derived from the untreated and TG-treated cells was immunoprecipitated with anti-ATF6α antibody followed by release of the immune complexes and reimmunoprecipitated (Re-IP) with anti-XBP1 antibody. The same Re-IP was also performed on the unbound supernatant fractions from the primary immunoprecipitation. While XBP1 antibody was able to immunoprecipitate the CENPF promoter (− 679 to − 488 and − 241 to − 78), subsequent Re-IPs of the eluted primary immunoprecipitates were able to bind the CENPF promoter (− 679 to − 488 and −241 to − 78) (Fig. 4c, d). Figure 4c, d showed that both XBP1 and ATF6*α* were bound to the CENPF promoter, apparently in the same complex. These experiments supported a model in which XBP1 and ATF6*α* act in a combinatorial fashion on the CENPF promoter.

### Mutagenesis analysis of XBP1 binding motifs involved in regulating the ER stress-induced transcriptional activity of the CENPF promoter

**Fig. 5:**
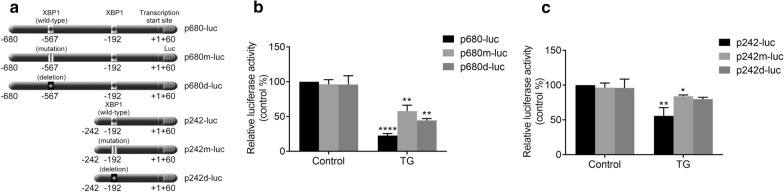
Mutagenesis analysis of XBP1 binding motifs involved in TG-induced transcriptional activity of the CENPF promoter. **a** Construction of CENPF promoter/luc vectors with XBP1 binding elements. Wild-type, mutant and deletion XBP1 binding elements (position − 567) are denoted as p680-luc, p680m-luc and p680d-luc, respectively. Wild-type, mutant and deletion XBP1 binding element (position − 192) are denoted by p242-luc, p242m-luc and p242d-luc, respectively. **b**, **c** Relative luciferase activities with or without TG treatment for 24 h. Measured luciferase activities were normalized to Renilla luciferase activities. Results are expressed as a percentage of the untreated control that is taken as 100%. Data are representative of 3 independent experiments. Data are presented as mean ± s.e.m. *P < 0.05, **P < 0.005, ***P < 0.001, ****P < 0.0001

To investigate whether the identified XBP1-binding sequences are required for the reduction of CENPF promoter activity induced by ER stress, we constructed mutant and deletion versions of p680-luc and p242-luc, named p680m-luc, p680d-luc, p242m-luc and p242d-luc, respectively (Fig. 5c). As shown in Fig. 5b, following the treatment with TG, p680-luc activity was decreased to 22% and the decrease was abolished by mutation or deletion of the XBP1 binding sequence (− 567). On the other hand, the activity of p1242-luc was decreased 56% of control in the presence of ER stress (Fig. 5c) and the decrease was also abolished by mutation or deletion of the XBP1 binding sequence (− 192) (Fig. 5c).

These results support the hypothesis that ER stress regulates the transcriptional activity of the CENPF gene via the XBP1-mediated trans-repression. The XBP1 binding sequences (− 567 and − 192) in the CENPF promoter play a modulatory role in this process.

### Overexpression of CENPF inhibits cell apoptosis and promotes cell proliferation in response to ER stress

**Fig. 6:**
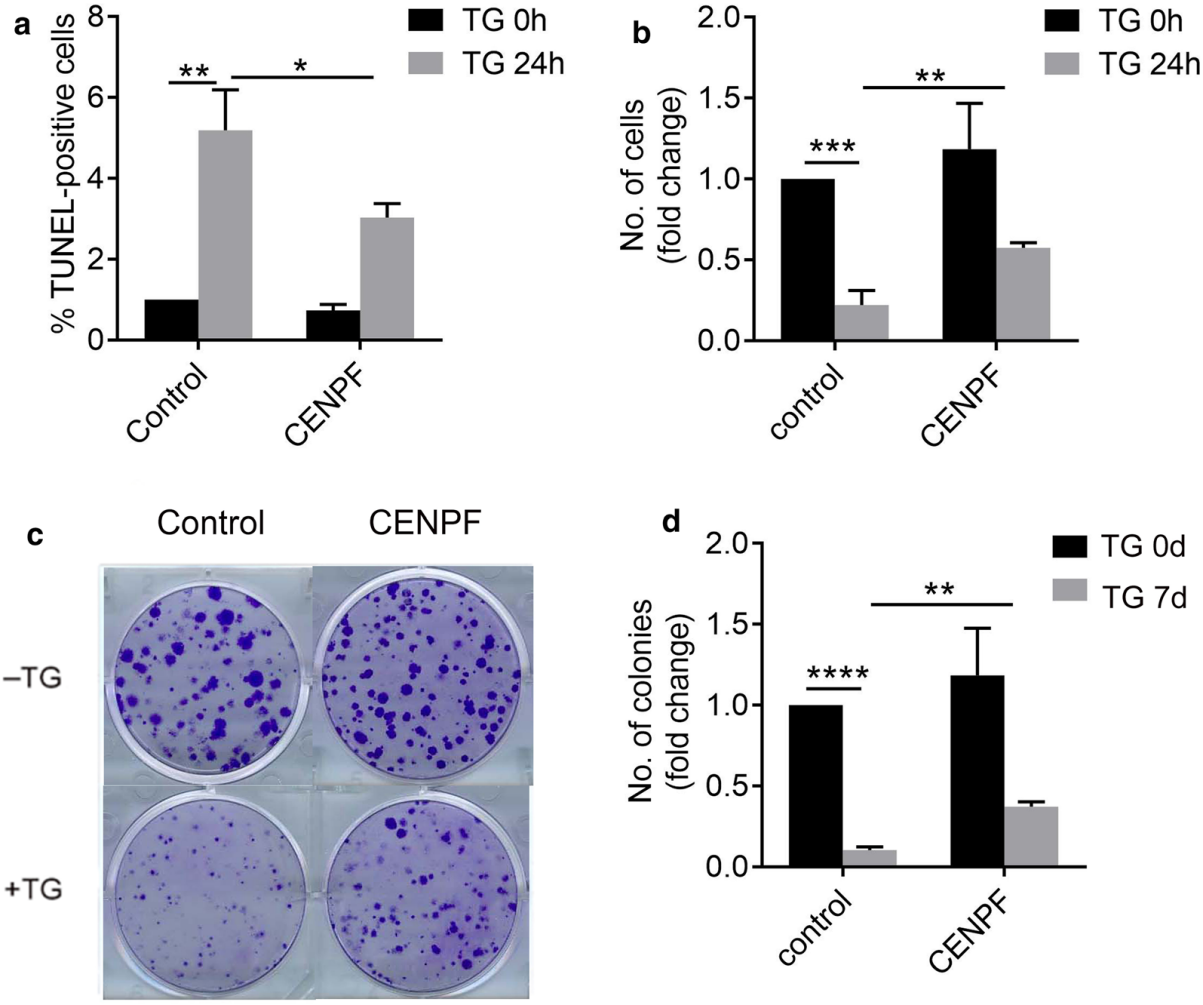
Overexpression of CENPF inhibits cell apoptosis and promotes cell proliferation in response to ER stress. **a** U2OS cells transfected with or without transient overexpression of CENPF were incubated with 1 μM TG for 24 h, and apoptotic cells were counted using a TUNEL assay. **b** U2OS cells transfected with or without transient overexpression of CENPF were incubated with or without TG for 24 h. Viable cells were counted using trypan blue exclusion. **c** U2OS cells transfected with control or CENPF plasmid were incubated with or without TG for 7 days. Colonies were stained with crystal violet and counted. Data are representative of 3 independent experiments. Data are presented as mean ± s.e.m. *P < 0.05, **P < 0.005, ***P < 0.001, ****P < 0.0001

Since high levels of ER stress activate cellular apoptosis, we asked whether CENPF could contribute to ER stress-induced apoptosis in U2OS cells. TG treatment of control cells increased apoptosis after 24 h, and the activity attenuated in overexpression of CENPF (Fig. 6a and Additional file 4: S4a). Furthermore, because TG treatment normally inhibits cell proliferation, we also asked whether CENPF overexpression could contribute to proliferation in U2OS cells along with blocking the effects of ER stress-induced apoptosis. The inhibition of cell proliferation by TG-induced ER stress was attenuated by overexpression of CENPF (Fig. 6b). We also obtained similar results when we examined clonogenic growth of U2OS cells after TG treatment. (Figure 6c and Additional file 4: S4b).

## Discussion

In this study, we demonstrated that ER stress plays a crucial role in regulating CENPF transcription and protein expression in human osteosarcoma cells. Importantly, we identify two XBP1 binding sequences at positions − 567 and −192 in the 5′-flanking region of the CENPF gene. Moreover, the results suggest that ER stress induced XBP1 binding affinity may arise, at least in part, via recruiting ATF6α, to decrease the CENPF transcription levels through the transcriptional inhibition of the CENPF gene promoter.

Recent studies have reported that the CENPF gene is transcriptionally responsive to a variety of signals, including DNA damage, mitotic stress, oxidative stress, or hypoxic stress [19–21]. Accordingly, we examined the effect of 3 different ER stress inducers on the regulation of CENPF mRNA and protein levels in different osteosarcoma cell lines. Our results demonstrate that treatment with each of these ER stress inducers dramatically decreased both CENPF mRNA and protein level as well as protein level in U2OS and MG-63 cells in a time-dependent manner. This led to our proposal that ER stress can have an important role in the regulation of CENPF gene expression.

We further examined which ER stress associated signaling pathway is involved in CENPF regulation. We showed IRE1α and ATF6*α*, rather than the PERK pathways are important for ER stress-mediated induction CENPF gene expression. In addition, we demonstrated that the XBP1 transcription factor is required for the ER stress-induced CENPF gene expression. Other transcription factors downstream of IRE1α signaling pathway, such as NF-κB [27], may also be involved in this process and thus are currently under investigation.

Promoter analysis and luciferase activity analyses were performed to further elucidate the molecular mechanisms underlying the ER stress-mediated regulation of the CENPF gene. Our results showed that the negative regulatory regions from − 679 to − 488 and from − 241 to − 78 in the CENPF promoter are important for sensitivity to ER stress, and that two putative XBP1 binding sites are located in these positions. Subsequently, we used ChIP assays to demonstrate a physical association of XBP1 with the CENPF promoter. Further use of the ChIP assay showed that specific XBP1 binding to the CENPF promoter is increased by induction of ER stress under in vivo conditions.

It must be noted that ER stress-mediated regulation of the CENPF promoter does not exclude other transcription factors (C/EBPα, Sp-1, GATA-1 etc.) known to be affected by ER stress which might also be involved in the CENPF expression regulation. This possibility may also explain why the mutant constructs p242m-luc exhibits minor changes in CENPF promoter activity (84% of untreated control) when exposed to TG. Such minor changes in promoter activity could reflect a cumulative effect of TG on ER stress-responsive transcription activators/repressors such as Sp-1 that bind to other sites in proximity to the XBP1 binding elements. Further studies are needed to identify other co-transcription factors which might form a complex with XBP1 or ATF6 to induce the transcription of CENPF gene.

Osteosarcoma is the most common bone malignancy, predominantly affecting adolescents and young adults. A complete understanding of molecular mechanisms underlying osteosarcoma tumorigenesis are still unclear. Our study demonstrates that CENPF represents an ER stress response gene that participates in XBP1 and ATF6 signaling pathway, inhibiting cell apoptosis and promoting cell proliferation in response to ER stress. However, there are still some limitations in the present study, in vivo studies are needed to clarify osteosarcoma cell apoptosis and proliferation caused by CENPF resulting from XBP1 and ATF6 signaling pathway. Moreover, the generalizability of this study will be explored. Our findings may help to achieve a better understanding of CENPF expression regulation in osteosarcoma progression.

## Conclusion

In summary, our results demonstrated that ER stress plays a crucial role in CENPF expression, and XBP1 may up-regulate DNA-binding affinities via recruiting ATF6α, to decrease the CENPF transcription levels under ER stress. Our findings also demonstrate that CENPF is a new ER stress response gene that participates in XBP1 and ATF6 signaling pathway to inhibit cell apoptosis and promote cell proliferation. These findings may help to achieve a better understanding of how control of CENPF expression is relevant to osteosarcoma progression and, further, how manipulation of CENPF might be used as a therapeutic approach to treating osteosarcoma.

## Supplementary information

**Additional file 1: Fig. S1.** CENPF expression is downregulated in osteosarcoma MG-63 cells in response to ER stress. MG-63 cells were treated with DMSO vehicle (control), BFA (1 μg/ml), TG (1 μM) or TM (2.5 μg/ml) for 0, 6 or 24 hours. CENPF mRNA levels were quantified by real-time reverse transcription-PCR (q-RT-PCR) and normalized to 18S RNA. (b) Changes in CENPF protein levels induced by BFA, TG and TM treatment in MG-63 cells. CENPF and GAPDH levels were determined by western blot analysis. Data are representative of 3 independent experiments. Data are shown mean ± s.e.m. *P < 0.05, **P < 0.005, ***P < 0.001, ****P < 0.0001.

**Additional file 2: Fig. S2.** q-RT-PCR analysis of different transcription factor mRNA level in U2OS cells with or without transient knockdown of CHOP, XBP1 and ATF4. Data are representative of 3 independent experiments. Data are presented as mean ± s.e.m. *P < 0.05, **P < 0.005, ***P < 0.001, ****P < 0.0001.

**Additional file 3: Fig. S3.**Nucleotide sequence of –840 to +60 sequence of the CENPF promoter (ENSG00000117724). Potential transcription factor binding motifs are boxed.

**Additional file 4: Fig. S4.**Western blotting analysis of CENPF plasmid expression. (a) U2OS cells transfected with or without transient overexpression of CENPF were incubated with 1 μM TG for 24 hours, and CENPF protein level was estimated by western blot. (b) U2OS cells transfected with control or CENPF plasmid were incubated with or without TG for 7 days. and CENPF protein level was estimated by western blot. Data are representative of 3 independent experiments. Data are presented as mean ± s.e.m. *P < 0.05, **P < 0.005, ***P < 0.001, ****P < 0.0001.

## Data Availability

The data are available from the corresponding author upon reasonable request.

## Abbreviations

XBP1: X-box binding protein 1
ATF6α: Activating transcription factor 6α
CENPF: Centromere protein F
ER: Endoplasmic reticulum
TG: Thapsigargin
BFA: Brefeldin A
TM: tunicamycin
ChIP: Chromatin immunoprecipitation
FOXM1: Forkhead box M1
UPR: Unfolded protein response
IRE1α: Inositol-requiring protein 1α
PERK: PRKR-like ER kinase
ERAD: ER-associated protein degradation
