# Supplementary figures and images for: XBP1 negatively regulates CENPF expression via recruiting ATF6α to the promoter during ER stress
Source: Cancer Cell Int. 2020 Sep 22;20:459. doi: 10.1186/s12935-020-01553-9 (PMC7507253; doi:10.1186/s12935-020-01553-9)

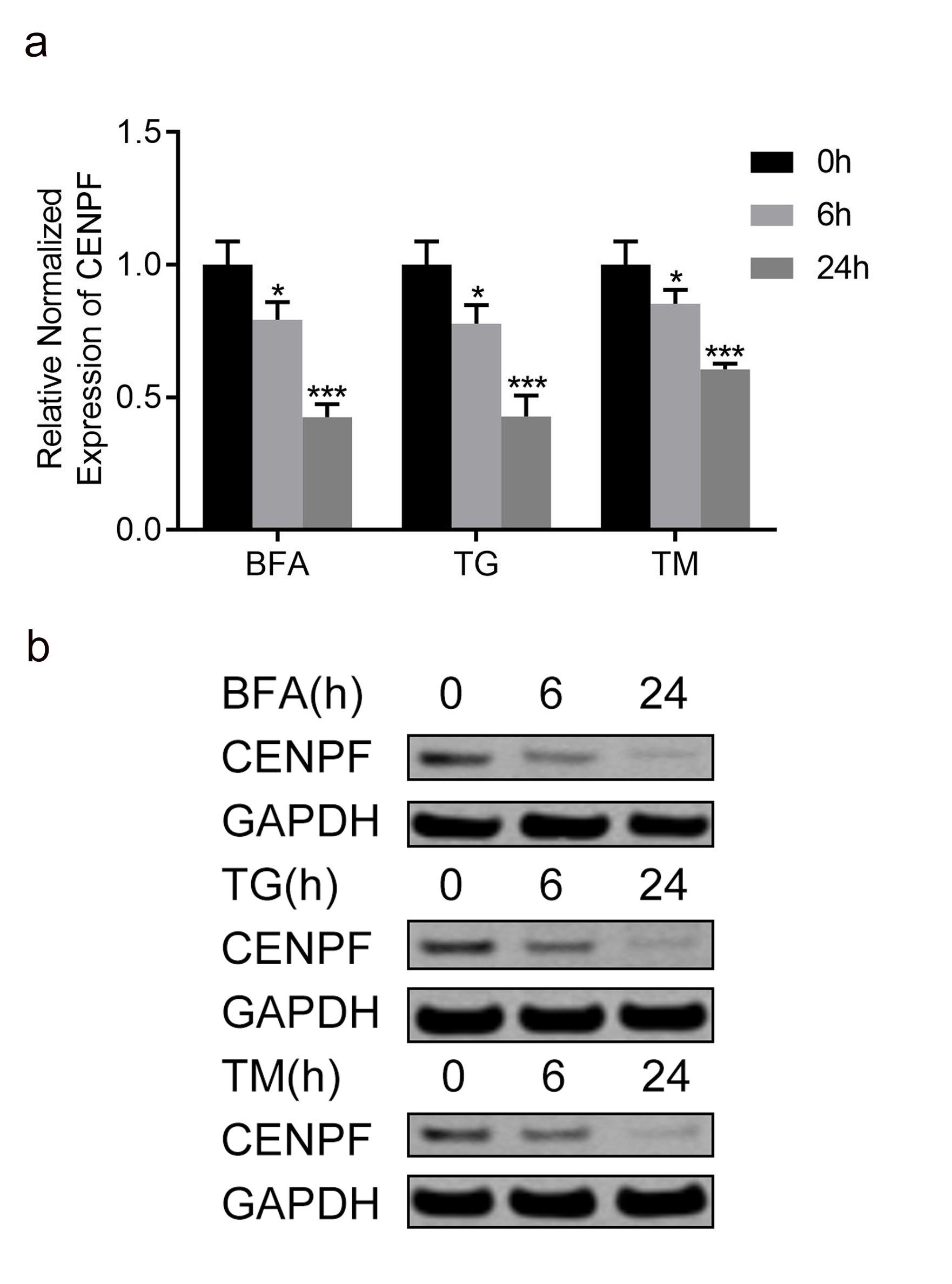

Supplement: Supplementary file 1 — Additional file 1: Fig. S1. CENPF expression is downregulated in osteosarcoma MG-63 cells in response to ER stress. MG-63 cells were treated with DMSO vehicle (control), BFA (1 μg/ml), TG (1 μM) or TM (2.5 μg/ml) for 0, 6 or 24 hours. CENPF mRNA levels were quantified by real-time reverse transcription-PCR (q-RT-PCR) and normalized to 18S RNA. (b) Changes in CENPF protein levels induced by BFA, TG and TM treatment in MG-63 cells. CENPF and GAPDH levels were determined by western blot analysis. Data are representative of 3 independent experiments. Data are shown mean ± s.e.m. *P < 0.05, **P < 0.005, ***P < 0.001, ****P < 0.0001. [file 12935_2020_1553_MOESM1_ESM.jpg]

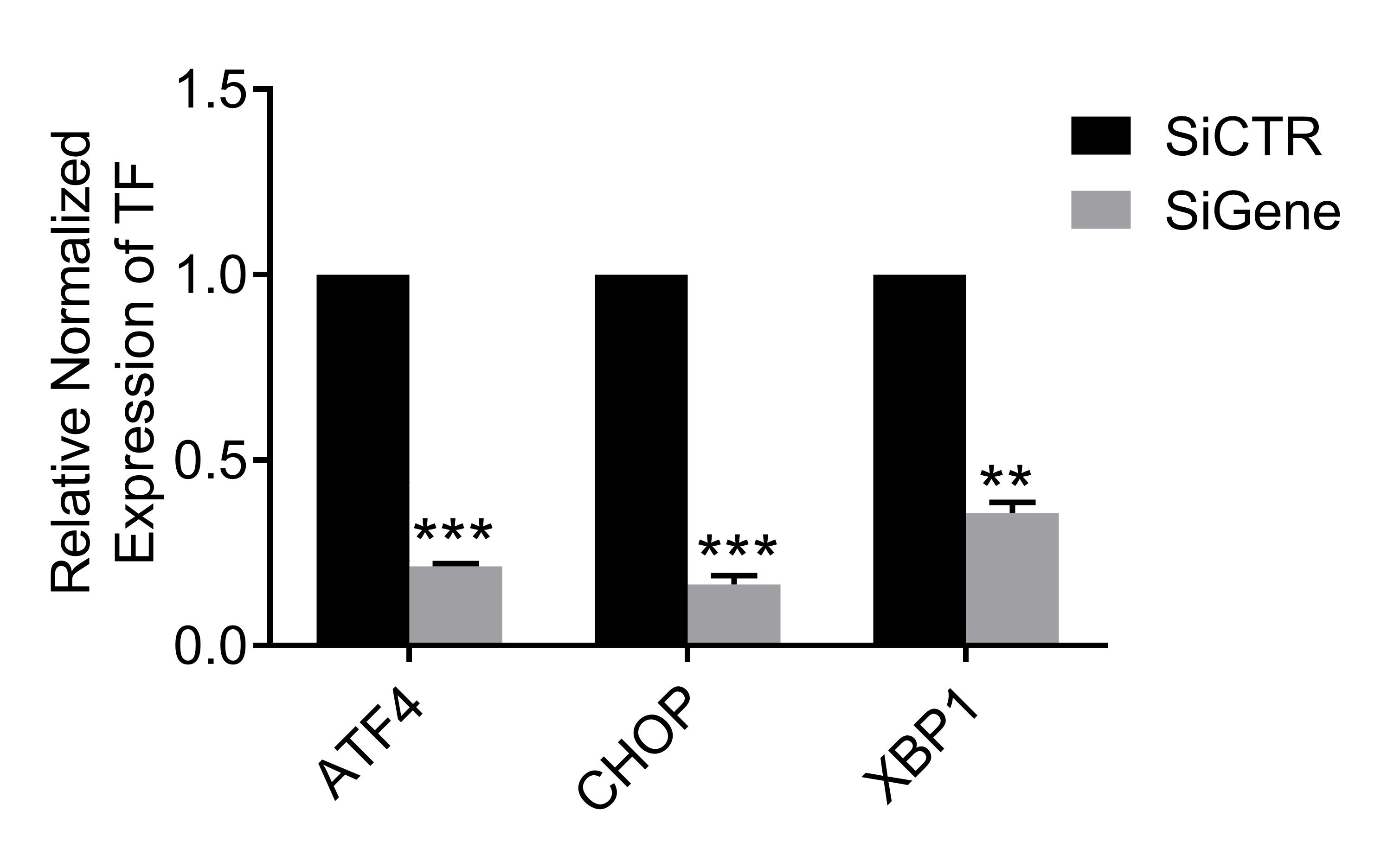

Supplement: Supplementary file 2 — Additional file 2: Fig. S2. q-RT-PCR analysis of different transcription factor mRNA level in U2OS cells with or without transient knockdown of CHOP, XBP1 and ATF4. Data are representative of 3 independent experiments. Data are presented as mean ± s.e.m. *P < 0.05, **P < 0.005, ***P < 0.001, ****P < 0.0001. [file 12935_2020_1553_MOESM2_ESM.jpg]

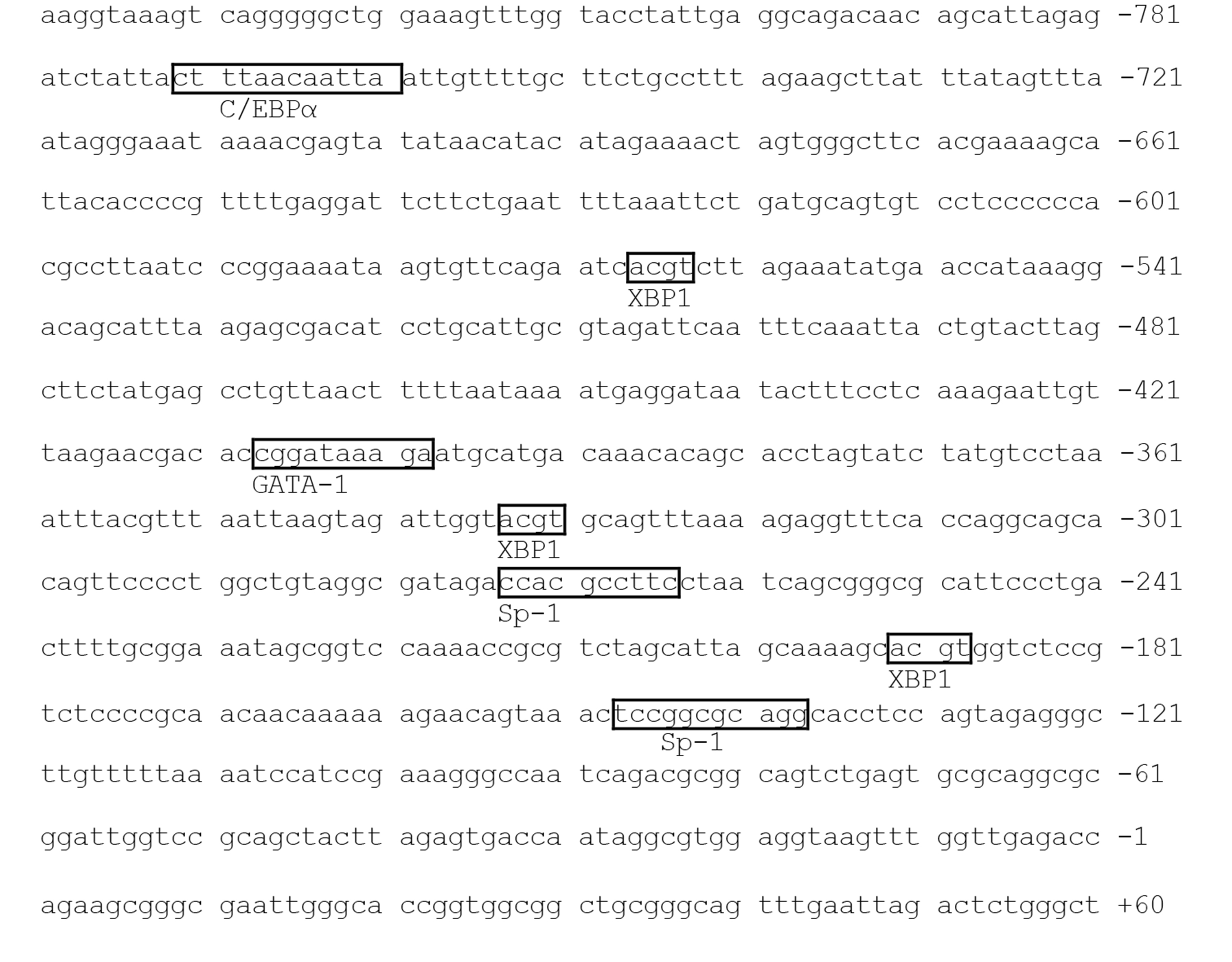

Supplement: Supplementary file 3 — Additional file 3: Fig. S3.Nucleotide sequence of –840 to +60 sequence of the CENPF promoter (ENSG00000117724). Potential transcription factor binding motifs are boxed. [file 12935_2020_1553_MOESM3_ESM.jpg]

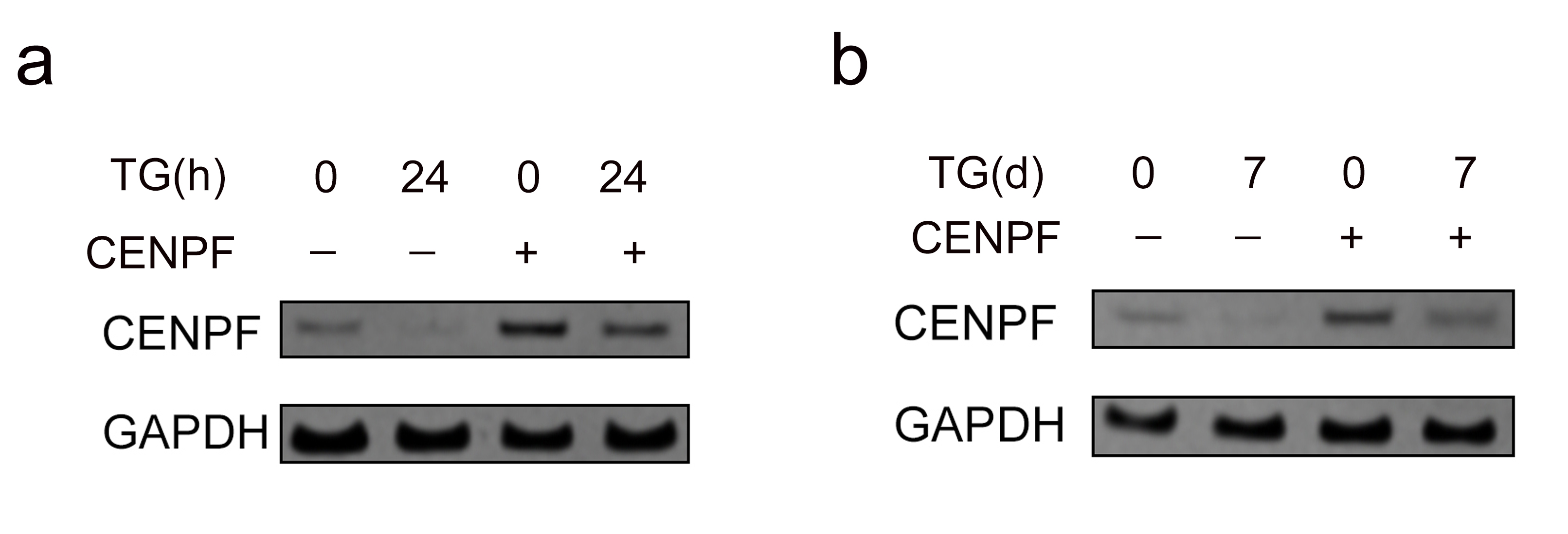

Supplement: Supplementary file 4 — Additional file 4: Fig. S4.Western blotting analysis of CENPF plasmid expression. (a) U2OS cells transfected with or without transient overexpression of CENPF were incubated with 1 μM TG for 24 hours, and CENPF protein level was estimated by western blot. (b) U2OS cells transfected with control or CENPF plasmid were incubated with or without TG for 7 days. and CENPF protein level was estimated by western blot. Data are representative of 3 independent experiments. Data are presented as mean ± s.e.m. *P < 0.05, **P < 0.005, ***P < 0.001, ****P < 0.0001. [file 12935_2020_1553_MOESM4_ESM.jpg]
